# Pneumomediastinum Following Heimlich Maneuver

**DOI:** 10.7759/cureus.94993

**Published:** 2025-10-20

**Authors:** Mohammed K Alsharef, Abdulrahman S Alozaymi, Mohammed F Alshehri, Mohammed M Alrishan

**Affiliations:** 1 Emergency Medicine Department, Ministry of the National Guard Health Affairs, Riyadh, SAU; 2 Emergency Medicine Department, Security Forces Hospital, Riyadh, SAU

**Keywords:** airway obstruction, case report, heimlich maneuver, pneumomediastinum, subcutaneous emphysema

## Abstract

The Heimlich maneuver is a universally accepted, life-saving technique that expels airway obstructions by generating elevated intrathoracic pressure through a sudden, upward-directed epigastric compression. While generally safe, complications can occur. Pneumomediastinum, the presence of air within the mediastinum, is an uncommon but significant potential complication that arises from alveolar rupture and air tracking along bronchovascular sheaths due to sudden pressure changes. We present the case of a 27-year-old man with no significant medical history who presented to the emergency department with chest pain and dyspnea after choking on a piece of meat. His father performed multiple Heimlich maneuvers until the obstruction was relieved. He subsequently developed sharp, retrosternal chest pain radiating to the back, dyspnea, and two episodes of blood-streaked vomiting. Physical examination revealed subcutaneous emphysema in the right anterior triangle of the neck. His vital signs were stable. A chest radiograph demonstrated pneumomediastinum and subcutaneous emphysema. The patient was kept nil per os and received oxygen via nasal cannula, intravenous fluids, and empirical intravenous antibiotics. Computed tomography imaging confirmed pneumomediastinum without evidence of esophageal rupture. He was admitted for observation, managed conservatively, and discharged home after two days without complications. Pneumomediastinum is a rare but significant complication of the Heimlich maneuver. Clinicians should maintain awareness of this risk, ensure early recognition through clinical assessment and imaging, and pursue appropriate conservative management to achieve favorable outcomes. Education on proper technique may help reduce the risk of such complications.

## Introduction

In 1974, Henry Heimlich first described his life-saving maneuver of applying abdominal pressure to the infradiaphragmatic area to expel inhaled food from the upper airways. The Heimlich maneuver generates elevated intrathoracic pressure through a sudden, upward-directed epigastric compression [1]. It is a universally accepted procedure for clearing a blocked airway [2] and is also recommended for removing inhaled foreign bodies in children who are choking [3].

The maneuver increases intrathoracic pressure, causing forced expiration of trapped intrapulmonary air and expulsion of the airway obstruction. Forced expiration against a closed glottis, especially in the setting of a true foreign body impaction in the upper aerodigestive tract, can lead to complications [4]. Reported adverse effects include vomiting and rib fractures. More severe complications include esophagogastric and jejunal ruptures [5-7]. In this report, we present the case of a 27-year-old man who developed pneumomediastinum as a complication of the Heimlich maneuver performed after choking on meat.

## Case presentation

A 27-year-old man with no known medical conditions or history of surgery presented to the emergency department with chest pain and dyspnea. He reported choking on a piece of meat while eating. His father performed the Heimlich maneuver multiple times until the obstruction was relieved. Soon afterward, he developed sharp, retrosternal chest pain radiating to the back, along with dyspnea and two episodes of vomiting mixed with blood spots.

On examination, the patient was alert, oriented, and not in distress. His systolic blood pressure was 139 mm Hg, diastolic blood pressure 89 mm Hg, mean arterial pressure 99 mm Hg, heart rate 95 beats/min, respiratory rate 18 breaths/min, and oxygen saturation 96% on room air. Physical examination revealed subcutaneous emphysema in the right anterior triangle of the neck. Chest auscultation findings and results of the throat and abdominal examinations were unremarkable. The electrocardiogram, complete blood count, electrolyte panel, and renal function tests were all within normal reference limits. The patient’s initial white blood cell count was within reference limits at 8.14 ×10⁹/L (reference range: 4-11 ×10⁹/L), with a normal neutrophil count (Table 1). These findings were not suggestive of an underlying infection or systemic inflammatory response, and there were no changes over the next 48 hours.

**Table 1:** Patient laboratory values ^a^Glucose (Glu2) reference ranges vary by collection context: fasting male 4.2–6.4 mmol/L; fasting female 3.9–6.1 mmol/L; random 3.9–7.7 mmol/L; pediatric 3.3–5.8 mmol/L. Critical ranges: pediatric <2.6 or >24.7 mmol/L; adult <2.6 or >26.9 mmol/L. ^b^Therapeutic guidance for anticoagulation: common INR targets 2.0–3.0 for most thromboembolic states; 2.5–3.5 for mechanical valves/recurrent embolism. aPTT heparin therapeutic range: 49–81 s (per lab note). ABO: ABO blood group system; ALT: alanine aminotransferase; aPTT: activated partial thromboplastin time; AST: aspartate aminotransferase; CO₂: carbon dioxide (bicarbonate); eGFR: estimated glomerular filtration rate; Glu2: second glucose measurement; INR: international normalized ratio; MCH: mean corpuscular hemoglobin; MCHC: mean corpuscular hemoglobin concentration; MCV: mean corpuscular volume; MPV: mean platelet volume; NRBC: nucleated red blood cells; RDW: red cell distribution width; Rh: Rhesus (D) antigen.

Analyte	Patient value	Reference range
Glucose (Glu2)	6.5 mmol/L	See context-specific ranges^a^
Basophils, absolute	0.02 ×10^9/L	0–0.1 ×10^9/L
Basophils, %	0.24%	—
Eosinophils, absolute	0.44 ×10^9/L	0.1–0.7 ×10^9/L
Eosinophils, %	5.41%	—
Neutrophils, %	54.30%	—
Lymphocytes, %	33.70%	—
Monocytes, absolute	0.52 ×10^9/L	0.1–1.1 ×10^9/L
Monocytes, %	6.33%	—
Neutrophils, absolute	4.41 ×10^9/L	2–7.5 ×10^9/L
Lymphocytes, absolute	2.75 ×10^9/L	1–4.4 ×10^9/L
Hemoglobin	164 g/L	135–180 g/L
White blood cells	8.14 ×10^9/L	4–11 ×10^9/L
Red blood cells	5.61 ×10^12/L	4.5–6.1 ×10^12/L
Hematocrit	0.504 L/L	0.42–0.54 L/L
MCV	89.8 fL	76–96 fL
MCH	29.3 pg	27–32 pg
MCHC	326 g/L	320–350 g/L
RDW	13.2%	11.5–14.5%
NRBC, absolute	0.00 ×10^9/L	0–3 ×10^9/L
Platelets	300 ×10^9/L	150–400 ×10^9/L
NRBC, %	0.00%	0–5%
MPV	10.5 fL	7.4–10.4 fL
INR	1.15	0.80–1.20^b^
Prothrombin time	12.40 s	9.38–12.34 s
aPTT	26.40 s	24.84–32.96 s
eGFR	136 mL/min/1.73 m²	≥60 mL/min/1.73 m²
Uric acid	387 µmol/L	220–450 µmol/L
Bilirubin, total	9.7 µmol/L	≤20.5 µmol/L
Alkaline phosphatase	63 U/L	40–150 U/L
Total protein	76 g/L	64–83 g/L
Phosphorus	1.23 mmol/L	0.74–1.52 mmol/L
Adjusted calcium	2.16 mmol/L	2.10–2.55 mmol/L
Potassium	4.4 mmol/L	3.5–5.1 mmol/L
Albumin	47 g/L	35–52 g/L
Magnesium	0.74 mmol/L	0.66–1.07 mmol/L
Sodium	140 mmol/L	136–145 mmol/L
Chloride	105 mmol/L	98–107 mmol/L
Creatinine	65 µmol/L	64–110 µmol/L
CO₂ (bicarbonate)	22 mmol/L	22–29 mmol/L
Glucose (random)	6.3 mmol/L	2.9–7.8 mmol/L
Serum urea nitrogen	3.4 mmol/L	3.2–7.4 mmol/L
Calcium (total)	2.30 mmol/L	2.10–2.55 mmol/L
ALT	21 U/L	5–55 U/L
AST	35 U/L	5–34 U/L
Anion gap	17 mmol/L	7–15 mmol/L
ABO group	AB	—
Rh (D antigen)	Negative	—
Antibody screen	Negative	—

**Figure 1:**
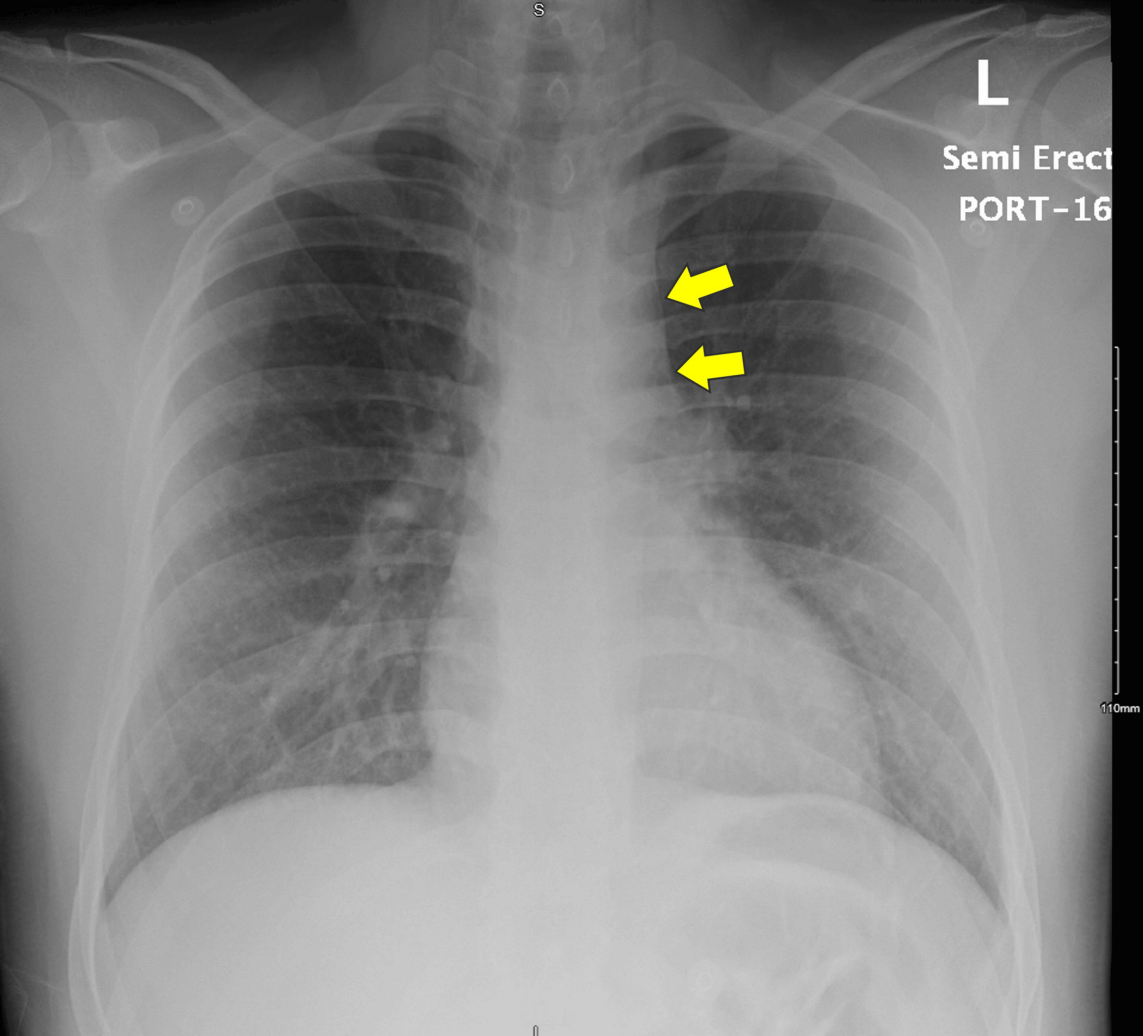
Portable chest radiograph showing pneumomediastinum (arrows)

A portable chest radiograph showed pneumomediastinum and neck subcutaneous emphysema (Figure 1). The patient was kept nil per os, placed on 1 L/min oxygen via nasal cannula, and started on intravenous fluids. Given the presence of pneumomediastinum and vomiting with blood, esophageal rupture was suspected. Piperacillin-tazobactam (Tazocin) 4.5 g intravenous was administered, and a computed tomography (CT) scan with contrast was ordered.

**Figure 2:**
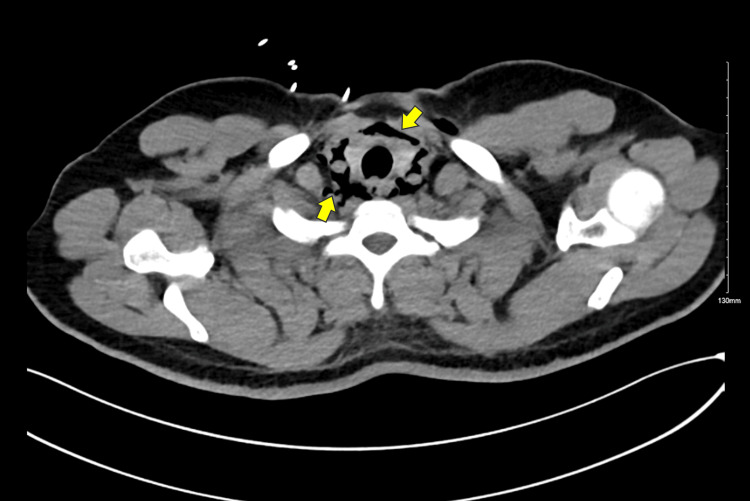
Axial chest CT with soft tissue window showing pneumomediastinum (arrows) CT: computed tomography

**Figure 3:**
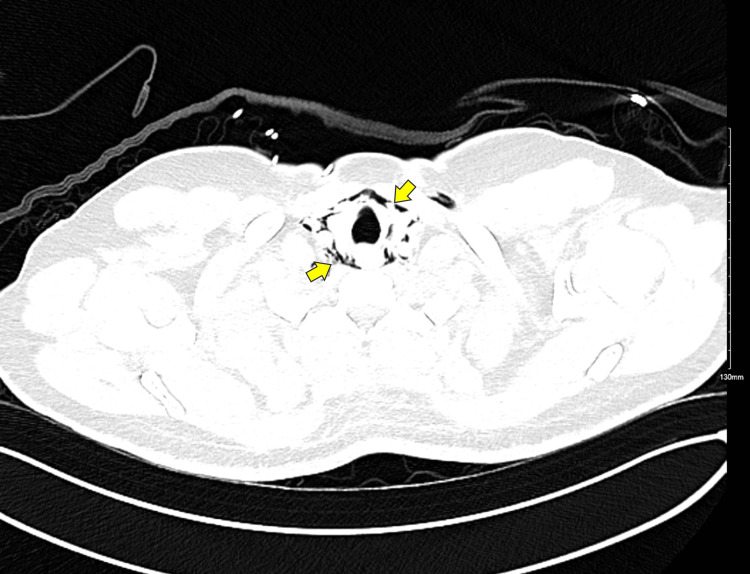
Axial chest CT with lung window highlighting pneumomediastinum (arrows) CT: computed tomography

The initial chest CT revealed pneumomediastinum without evidence of esophageal perforation (Figure 2). To further evaluate the possibility of an esophageal injury, a follow-up chest CT with oral contrast was conducted the following day (Figure 3). This subsequent CT also showed no signs of contrast leakage or other findings suggestive of esophageal perforation, effectively ruling out this diagnosis.

The patient was discharged in stable condition and returned for follow-up five days later. At that time, he was completely asymptomatic with no chest pain, dyspnea, or dysphagia. Clinical examination results were unremarkable, and he needed no further intervention.

## Discussion

Pneumomediastinum, also known as mediastinal emphysema, refers to the presence of air within the mediastinum. It can occur spontaneously or secondary to factors such as trauma, infections, foreign body aspiration with air trapping, or as a complication of forceful maneuvers such as the Valsalva maneuver [8,9].

The Heimlich maneuver generates sudden, forceful abdominal pressure, increasing intra-abdominal and intrathoracic pressures to expel a foreign body from the airway. This sudden pressure rise can cause alveolar rupture, allowing air to escape into the interstitial space and track along the bronchovascular sheaths into the mediastinum. This mechanism, known as the Macklin effect, explains the pathogenesis of pneumomediastinum following the Heimlich maneuver [10].

Several complications of the Heimlich maneuver have been reported, including pneumomediastinum, gastric rupture, abdominal aortic thrombosis, aortic valve rupture, aortic dissection, and pneumothorax [11]. Other complications of the Heimlich maneuver can include esophageal rupture, diaphragmatic rupture or hernia, hepatic rupture, pancreatic transection, mesenteric laceration, jejunal rupture, splenic rupture, cholesterol embolization, arterial occlusion, myocardial injury, rotator cuff tears, and thoracic vertebral fractures [11]. Pneumomediastinum has been specifically described in two previous case reports from 1979 and 1989 [2,12].

The risk of complications during the Heimlich maneuver depends on the experience of the person performing it, the number of attempts, the patient’s comorbidities, and age [11]. Older adults are at increased risk of choking due to neuromuscular disorders, age-related changes in the nervous system, muscular dystrophies, and dental problems that impair swallowing. Choking episodes often result from oropharyngeal abnormalities such as impaired laryngeal closure, failure of bolus containment, dissociation of the transitional phase, and ineffective bolus transport [13].

The Heimlich maneuver is more frequently used in older patients. Excessive force during repeated abdominal thrusts may cause injuries to internal organs, making the maneuver’s “quick upward thrust” its weakest link [14]. Repeated Heimlich maneuvers may dislodge the foreign body into the trachea, contributing to rapid development of severe subcutaneous emphysema, pneumomediastinum, and even pneumopericardium [3].

Subcutaneous emphysema typically resolves spontaneously within 3 to 10 days. Surgical drainage using an easy-flow, Penrose, or corrugated rubber drain may be required if emphysema is extensive, as tight closure without a drain can trap air in the subcutaneous planes. Oral feeding should be resumed only after confirmation of pharyngeal or esophageal integrity, typically with a barium swallow study, and after resolution of inflammatory markers. While surgery is the usual treatment for pharyngeal or esophageal perforation, some reports describe successful medical management without major complications [15-17].

## Conclusions

This case report highlights pneumomediastinum as a rare but significant complication of the Heimlich maneuver. Although the maneuver is life-saving, clinicians should remain aware of its potential risks. Prompt recognition through careful clinical assessment and appropriate imaging, followed by conservative management, typically results in a favorable outcome. Increased awareness and education about these risks can help minimize complications and improve patient safety.
